# In a Subgroup of High-Risk Asians, Telmisartan Was Non-Inferior to Ramipril and Better Tolerated in the Prevention of Cardiovascular Events

**DOI:** 10.1371/journal.pone.0013694

**Published:** 2010-12-21

**Authors:** Antonio L. Dans, Koon Teo, Peggy Gao, Jyh-Hong Chen, Kim Jae-Hyung, Khalid Yusoff, Suphachai Chaithiraphan, Jun Zhu, Liu Lisheng, Salim Yusuf, for the ONTARGET INVESTIGATORS

**Affiliations:** 1 University of the Philippines College of Medicine, Manila, Philippines; 2 Population Health Research Institute, McMaster University, Hamilton, Canada; 3 National Cheng Kung University Medical College, Tainan, Taiwan; 4 St. Paul's Hospital, The Catholic University of Korea, Seoul, Korea; 5 Universiti Teknologi MARA, Shah Alam, Malaysia; 6 Chaophya Hospital, Bangkok, Thailand; 7 FuWai Hospital, Chinese Academy of Medical Science, Beijing, China; Maastricht University, Netherlands

## Abstract

**Background and Objectives:**

Results of the recently published ONTARGET study (The Ongoing Telmisartan Alone and in Combination with Ramipril Global Endpoint Trial) showed that telmisartan (80 mg/day) was non-inferior to ramipril (10 mg/day) in reducing cardiovascular events. Clinicians in Asia doubt tolerability of these doses for their patients. We therefore analyzed data from this study and a parallel study TRANSCEND (Telmisartan Randomized Assessment Study in ACE Intolerant Subjects with Cardiovascular Disease). Our objectives were to compare Asians and non-Asians with respect to the following:

1) Effectiveness of telmisartan vs. ramipril in reducing cardiovascular events;
2) Proportions who reached the full dose of telmisartan, ramipril or placebo; and
3) Proportions of overall discontinuations, and discontinuations due to adverse effects.

**Method:**

The ONTARGET study randomized 25,620 patients at risk of cardiovascular events to ramipril, telmisartan, or their combination. The primary composite endpoint was death caused by cardiovascular disease, acute MI, stroke, and hospitalization because of congestive heart failure. TRANSCEND randomized 5926 high-risk patients with a history of intolerance to ACE-inhibitors to telmisartan or placebo. The primary outcome was the same. In this substudy, we compared Asians and non-Asians as to how well they tolerated telmisartan (given in both studies) and ramipril (given in ONTARGET).

**Results:**

1) Telmisartan was non-inferior to ramipril in lowering the primary endpoint among Asians (RR = 0.92; 95% CI: 0.74, 1.13); 2) more Asians achieved the full dose of either drug; 3) less withdrew (overall); and 4) less withdrew for adverse effects. Furthermore, telmisartan was better tolerated than ramipril. This advantage was greater among Asians.

**Conclusion and Significance:**

Although Asians had lower BMI than non-Asians, Asians tolerated both drugs better. Regulatory agencies require reporting of safety and effectiveness data by ethnicity, but few comply with this requirement. This study shows that safety data in ethnic subgroups can help assess applicability of results to specific populations.

**Trial Registration:**

ClinicalTrials.gov NCT00153101

## Introduction

Angiotensin-converting enzyme (ACE) inhibitors reduce cardiovascular morbidity and mortality among patients at risk for these events [1], [2]. However, studies also show a high residual rate of cardiovascular events despite treatment, and a significant incidence of intolerance to these drugs. These problems have led to interest in the role of Angiotensin Receptor Blockers (ARBs), either as supplements to, or replacements for ACE inhibitors [3].

The ONTARGET and TRANSCEND studies are 2 of the largest trials conducted to clarify the effect of ARBs on cardiovascular morbidity and mortality among patients at risk. In the ONTARGET study, 25,620 patients at risk of cardiovascular events (but no heart failure) were randomized to receive the ACE-inhibitor ramipril, the ARB telmisartan, or their combination. The primary composite endpoint was death caused by cardiovascular disease, acute MI, stroke, or hospitalization because of congestive heart failure. Results showed that telmisartan was non-inferior to ramipril in reducing these cardiovascular events among patients at risk [4]. Telmisartan was also better tolerated. Combining the 2 drugs showed no added advantage.

In TRANSCEND, the effect of telmisartan was compared to placebo, among 5926 patients at risk, who were intolerant to ACE-inhibitors. The primary outcome was the same as in the ONTARGET study. Results showed that the effect of telmisartan on the primary outcome was of borderline significance, probably because of an unexpectedly lower number of outcome events [5]. However, a pre-specified analysis combining these results with another study on telmisartan, the PRoFESS trial [6] (Prevention Regimen for Effectively Avoiding Second Strokes), showed a significant reduction in events, especially with more prolonged treatment.

Although these studies were conducted in 733 centers in 40 countries and regions, there is concern about the applicability of trial results to specific populations [7]. Both ONTARGET and TRANSCEND titrated telmisartan to a dose of 80 mg/day but studies in Asia show that lower doses are prescribed at least 75% of the time [8]. For ramipril, the target dose was 10 mg/day, but lower doses are prescribed 85% of the time in the region [8]. It is not surprising therefore, that in presentations of this study at numerous meetings in Asia, the most common question was whether Asians tolerated the drugs as well as non-Asians.

Many journals require race or ethnicity-specific data when reporting outcomes of epidemiologic studies or clinic trials. Because of space constraints however, this is rarely done [9]. The main goal of this paper, therefore, is to take a detailed look at the tolerability and safety of telmisartan and ramipril, comparing Asians and non-Asians in ONTARGET and TRANSCEND. Its specific objectives are:

1. To compare Asians and non-Asians with respect to the effectiveness of telmisartan vs. ramipril in reducing cardiovascular events in high risk patients;
2. To compare the proportions of Asians and non-Asians who reached the full dose of telmisartan, ramipril or placebo in both studies; and
3. To compare the proportions of overall discontinuations, and discontinuations due to adverse effects, between Asians and non-Asians in both studies.

In comparing Asians and Non-Asians, we decided to focus this report on the tolerability of telmisartan vs. ramipril (ONTARGET) and telmisartan vs. placebo (TRANSCEND). Tolerability of the combination of ramipril and telmisartan will not be examined because results of the ONTAGET study showed an increase in adverse events in this arm.

## Methods

The protocol was approved by regulatory authorities and the ethics review committee at each participating institution. Informed consent was obtained from every patient. Details of the methodology of ONTARGET and TRANSCEND have been previously reported [3]. Briefly, in ONTARGET, we enrolled patients aged 55 or more, if they had coronary, peripheral, or cerebrovascular disease or diabetes with end-organ damage (n = 25,620). Patients with the same characteristics but intolerant to ACE inhibitors were randomly assigned to receive either telmisartan or placebo in the TRANSCEND study (n = 5926) [4]. Both studies were double-blind, with a median follow-up of 56 months.

Telmisartan was titrated to a full dose of 80 mg in both ONTARGET and TRANSCEND. In the ONTARGET study, ramipril was titrated to a full dose of 10 mg per day. The main outcome was a composite of death from cardiovascular causes, myocardial infarction, stroke, or hospitalization for heart failure. The key secondary outcome was a composite of death from cardiovascular causes, myocardial infarction, or stroke, which was the primary outcome in the Heart Outcomes Prevention Evaluation (HOPE) trial [1]. National coordinators and clinical monitors supervised the recruitment and follow up of patients by investigators at 733 centers in 40 countries and regions. All outcomes were reviewed by a central adjudicating committee whose members were unaware of study-group assignments. An independent data and safety monitoring board reviewed all serious adverse events.

Information on ethnicity was based on self-reports during recruitment. For the purposes of this paper, Asian ethnicity included the following:

1. South Asian (India, Sri Lanka, Pakistan, Bangladesh, Afghanistan, Nepal),
2. Chinese (China, Hong Kong, Taiwan),
3. Japanese,
4. Malay, or
5. Other Asian (Korea, Papua New Guinea, Thailand, Philippines, Indonesia, Vietnam, Cambodia, Laos, Myanmar/Burma, Bhutan).

### Statistical Analysis

Analysis was done using SAS version 8.2 (SAS Institute, Cary, NC). The primary analysis used a time-to-event approach and included all Asian and non-Asian participants who were randomized to ramipril and telmisartan arms in ONTARGET. Treatment comparisons between telmisartan and ramipril using the predefined non-inferiority margin 1.13, and between Asians and non-Asians with regard to time-to-event related data (time to occurrence of first event) were shown as hazard ratios with 95% confidence intervals. A Cox regression model, with factors for treatment, Asian ethnicity, and interactions, was used to examine the treatment effect among Asian and non-Asian patients. Treatment comparisons (telmisartan vs. ramipril in ONTARGET, telmisartan vs. placebo in TRANSCEND) with regard to tolerability were done using the χ^2^ test by comparing discontinuations and the proportion achieving the full dose. The Breslow-Day method was used to test homogeneity among Asian and non-Asian patients. All reported p-values (other than for non-inferiority) were two sided. P<0.05 was considered significant.

## Results

Overall, Asians accounted for 3521 out of 25,620 patients randomized in ONTARGET, and 1261 out of 5926 patients randomized in TRANSCEND. Table 1 compares key baseline characteristics of Asians and Non-Asians in both studies. Risk factors for atherosclerosis were about the same in terms of prevalence of hypertension and smoking. Average age, blood pressure, blood glucose, and lipid levels were likewise similar. Asians tended to have lower BMI, a higher incidence of diabetes and previous stroke, slightly less coronary disease, more angioplasties, and less bypass surgeries. Statins and diuretics were used less often, while calcium channel blocker use was more common among Asians.

**Table 1:** Baseline Characteristics of Asians and Non-Asians in the ONTARGET and TRANSCEND studies.

	ONTARGET		TRANSCEND	
	ASIANS	NON-ASIANS	ASIANS	NON-ASIANS
	(n = 3521)	(n = 22,092*)	(n = 1261)	(n = 4665)
Mean age (years)	65.48	66.58	65.64	67.2
Male Sex (%)	73.7	73.3	59.7	56.3
Hypertension (%)	69.1	68.7	73.4	77.2
Diabetes (%)	43.1	36.6	41.7	34.1
Stroke or TIA (%)	30.3	19.3	34.2	18.7
Coronary disease (%)	71.1	75.1	70.1	75.8
Current Smokers (%)	12.3	12.6	10.6	9.6
Mean Systolic BP (mmHg)	141	142	140	141
Mean BMI (KG/m^2^)	25.5	28.51	25.9	28.73
Mean cholesterol (mmol/Li)	4.97	4.94	4.99	5.11
Mean HDL (mmol/Li)	1.25	1.26	1.27	1.28
Mean LDL (mmol/Li)	2.95	2.92	2.98	3.04
Mean triglycerides (mmol/Li)	1.73	1.73	1.77	1.78
Fasting glucose (mmol/Li)	6.73	6.67	6.55	6.49
Prior coronary bypass (%)	11.0	23.9	10.3	21.2
Prior PTCA (%)	35.1	28.1	32.4	24.5
Statin use (%)	49.5	63.5	48.4	57.1
Beta-Blocker use (%)	52.2	57.7	52.0	60.0
Aspirin use (%)	77.6	75.4	73.5	75.0
Thienopyridine use (%)	9.1	11.3	10.7	10.7
Diuretic use (%)	18.1	29.5	22.5	35.8
Calcium blocker use (%)	43.9	31.3	50.0	37.5

*7 patients with missing ethnicity.

BP = blood pressure; BMI = body mass index; HDL = high density lipoprotein cholesterol; LDL = low density lipoprotein cholesterol; PTCA = percutaneous transluminal coronary angioplasty.

### Cardiovascular Events

Table 2 shows the incidence of cardiovascular events on ramipril and telmisartan. In the overall study result, telmisartan was proven non-inferior to ramipril (HR = 1.01; 95% CI: 0.94–1.09). In this report, we show that this was a consistent result in the subgroup of Asians (HR = 0.92; 95% CI:0.74–1.13) as well as non-Asians (HR = 1.03; 95% CI:0.95–1.11). As can be seen in Table 2, there was no difference between these 2 groups in terms of hazard ratio for the primary composite outcome.

**Table 2:** Risk of the primary outcome (cardiovascular death, MI, stroke or admission for CHF) on Ramipril and Telmisartan, comparing Asians and Non-Asians in the ONTARGET Study.

	Overall	Asian	Non-Asian	p-value†
Ramipril	1412 (16.46%)	190 (16.07%)	1221 (16.52%)	0.775
Telmisartan	1423 (16.66%)	171 (14.59%)	1252 (16.99%)	0.082
HR (95% CI)*	1.01 (0.94–1.09)	0.92 (0.74–1.13)	1.03 (0.95–1.11)	0.305§
p-value¶	0.004	0.046	0.020	-

*Risk in telmisartan/ramipril group.

¶ p-value (telmisartan vs. ramipril) based on non-inferiority margin 1.13.

† p-value (asians vs. non-asians).

§ p for interaction.

Absolute rates of the composite outcome were generally the same, with slightly less events among Asians on telmisartan (HR = 0.87; 95% CI:0.74,1.02), but this did not reach statistical significance (p = 0.082). A more detailed comparison of the individual rates of cardiovascular death, MI, stroke or admission for CHF was also performed. This showed that overall, in the ONTARGET study, strokes were more common among Asians (5.9% vs. 4.3%, HR = 1.4; 95% CI:1.17–1.69; p = 0.003), while MI was more common among non-Asians (5.2% vs. 3.8%; HR = 1.35; 95% CI:1.09,1.67; p = 0.008). These differences persisted even after adjustment for differences in baseline characteristics (p = 0.042 for the difference in stroke, and p = 0.014 for the difference in the incidence of MI).

### Proportion Achieving Full Dose

Overall, a slightly lower proportion of patients achieved full dose ramipril (10 mg) than telmisartan (80 mg) in the ONTARGET study (74.8% vs. 79.8% respectively, p<0.0001). In both ONTARGET and TRANSCEND significantly more Asians than non-Asians achieved the target doses of ramipril and telmisartan (Table 3).

**Table 3:** Percent of Patients Achieving Full Dose Ramipril, Telmisartan or placebo at the end of the ONTARGET and TRANSCEND studies.

	Overall	Asians	Non-Asians	p-value†
ONTARGET				
Ramipril	5730 (74.82%)	826 (77.92%)	4903 (74.32%)	0.012
Telmisartan	6103 (79.82%)	922 (87.56%)	5180 (78.60%)	0.0001
RR (95% CI)*	1.08(1.05,1.09)	1.12(1.08,1.17)	1.06(1.04,1.08)	0.0003§
p-value¶	<0.0001	<0.0001	<0.0001	-
TRANSCEND				
Placebo	2088 (79.09%)	501 (86.68%)	1587 (76.96%)	<0.0001
Telmisartan	2086 (79.44%)	500 (86.36%)	1586 (77.48%)	<0.0001
RR (95% CI)**	1.00(0.98,1.03)	1.00(0.95,1.04)	1.01(0.97,1.04)	0.762§
p-valueδ	0.757	0.872	0.694	-

*Risk in telmisartan/ramipril group.

¶ p-value (telmisartan vs. ramipril).

**Risk in telmisartan/placebo group.

δ p-value (telmisartan vs. placebo).

† p-value (asians vs. non-asians).

§ p for interaction.

There was also a higher proportion of Asians achieving full-dose placebo in the TRANSCEND study, suggesting that Asians, were simply more compliant than non-Asians. However, this does not explain why, in ONTARGET, the difference favoring telmisartan over ramipril was significantly greater among Asians than non-Asians (p = 0.0003).

### Withdrawals

Table 4 shows that overall, in the ONTARGET study, there were more discontinuations in the ramipril group than in the telmisartan group (RR = 0.95; 95% CI: 0.90,1.00). There was no indication that Asians could not tolerate the doses at which either drug was given. On the contrary, there were significantly more discontinuations among non-Asians on either ramipril or telmisartan.

**Table 4:** Overall Discontinuations in Percent, in ONTARGET and TRANSCEND.

	Overall	Asians	Non-Asians	p-value†
ONTARGET Study				
Ramipril	2121 (24.73%)	235 (19.88%)	1885 (25.50%)	<0.0001
Telmisartan	2000 (23.41%)	169 (14.42%)	1829 (24.83%)	<0.0001
RR (95% CI)*	0.95 (0.90,1.00)	0.73 (0.61,0.87)	0.97 (0.92,1.03)	0.003§
p-value¶	0.044	0.0004	0.346	-
TRANSCEND Study				
Placebo	705 (23.72%)	82 (13.14%)	623 (26.53%)	<0.0001
Telmisartan	639 (21.63%)	83 (13.03%)	556 (24.00%)	<0.0001
RR (95% CI)**	0.91 (0.83,1.00)	0.99(0.75,1.32)	0.9(0.82,1.00)	0.489§
p-valueδ	0.055	0.953	0.046	-

*Risk in telmisartan/ramipril group.

¶ p-value (telmisartan vs. ramipril).

** Risk in telmisartan/placebo group.

δ p-value (telmisartan vs. placebo).

† p-value (asians vs. non-asians).

§ p for interaction.

In the TRANSCEND study, the lower rates of discontinuation among Asians on placebo suggests that Asians were simply more tolerant than non-Asians. However ONTARGET shows that the reduction in discontinuations with telmisartan compared to ramipril was more marked in Asians than in non-Asians (p = 0.003). This is consistent with the results on proportions of patients achieving full dose.

Table 5 tells a similar story, looking at permanent discontinuations that were specifically caused by side effects. There was no significant difference between Asians and non-Asians with respect to side effects from ramipril (p = 0.74). However, Asians tolerated telmisartan better than non-Asians (p = 0.0001 for ONTARGET, and p<0.0001 for TRANSCEND).

**Table 5:** Percent Permanent Discontinuations Because of Side effects in ONTARGET and TRANSCEND.

	Overall	Asians	Non-Asians	p-value†
ONTARGET Study				
Ramipril	1005(11.72%)	135(11.42%)	869 (11.76%)	0.740
Telmisartan	835(9.78%)	77 (6.57%)	757 (10.28%)	0.0001
RR (95% CI)*	0.83(0.76,0.91)	0.58(0.44,0.75)	0.87 (0.80,0.96)	0.004§
p-value¶	<0.0001	<0.0001	0.004	-
TRANSCEND Study				
Placebo	163 (5.49%)	18 (2.89%)	145 (6.18%)	0.001
Telmisartan	214 (7.24%)	22(3.45%)	192 (8.29%)	<0.0001
RR (95% CI)**	1.32 (1.08,1.61)	1.2(0.65,2.21)	1.34(1.09,1.65)	0.702§
p-valueδ	0.006	0.564	0.005	-

*Risk in telmisartan/ramipril group.

¶ p-value (telmisartan vs. ramipril).

** Risk in telmisartan/placebo group.

δ p-value (telmisartan vs. placebo).

† p-value (asians vs. non-asians).

§ p for interaction.

We also looked at differences in rates of discontinuation for specific reasons. Except for the higher incidence of cough among Asians (table 6) and the lower incidence of hypotension, we found no difference between Asians and non-Asians. Overall, in ONTARGET, the relative benefit from use of telmisartan showed a reduction in discontinuations because of cough (RR = 0.26; 95% CI: 0.21, 0.33). This was about the same in Asians and non-Asians, but the absolute risk of cough was higher in Asians. In the Ramipril group, 6.1% of Asians stopped because of cough, compared to only 3.9% of non-Asians (p<0.001). The risk of discontinuation due to hypotension in Asians was lower than in non-Asians (1.3% vs. 3.3%, p<0.0001 in ONTARGET and 0.2% vs. 0.9%, p = 0.016 in TRANSCEND). Despite these differences, there were more overall discontinuations among non-Asians. There were no significant differences in the other specific reasons for discontinuation such as syncope or angioedema.

**Table 6:** Percent Discontinuations Because of Cough in the ONTARGET Study.

	Overall	Asians	Non-Asians	p-value†
Ramipril	360 (4.2%)	72 (6.1%)	288(3.9%)	<0.001
Telmisartan	93(1.1%)	17(1.45%)	76 (1.03%)	0.200
RR (95% CI)*	0.26 (0.21,0.33)	0.24 (0.14,0.4)	0.26 (0.21,0.34)	0.679§
p-valueδ	<0.0001	<0.0001	<0.0001	

*Risk in telmisartan/ramipril group.

δ p-value (telmisartan vs. ramipril).

† p-value (asians vs. non-asians).

§ p for interaction.

## Discussion

Telmisartan was non-inferior to ramipril in lowering the risk of cardiovascular death, stroke, MI or admission for CHF, and was slightly better tolerated. This was a consistent finding among Asians and non-Asians in the ONTARGET study. The similarity in absolute event rates between Asians and non-Asians reassures us that there were no adverse differences related to the primary outcomes.

Our measures of tolerability included the proportion of patients who achieved the full dose at the study's end, and the proportion who had to discontinue permanently. Both measures showed an interesting trend, that Asians were generally more tolerant, even when they were on placebo. Possible explanations include the following:

1. Asians are more tolerant and compliant to their prescribed medications in general;
2. Most principal investigators in the participating centers from the Asian countries and regions were also the responsible physician for their patients. This may have provided supportive reassurance when minor side effects develop.

These differences do not affect the validity of the noted differences between telmisartan and ramipril in ONTARGET, and between telmisartan and placebo in TRANSCEND, because both trials were double blind, and the proportion of Asians was equal in all treatment groups. Furthermore, while these differences may explain the higher tolerance of Asians for both ramipril and telmisartan, they do not explain why the advantage of telmisartan over ramipril was greater among Asians than non-Asians (tables 3 and 4). This observation raises the possibility that there may be ethnic differences in the relative tolerance to these 2 classes of drugs in RAA inhibition. Ethnic differences in drug tolerability are well documented, especially in the case of cardiovascular drugs [10]. These differences may be related to cultural factors such as diet [11] or compliance, or genetic factors such as drug metabolism or RAA activity [12]–[14].

Our findings are consistent with previous literature showing that cough is more common among Asians [15]. Asian ethnicity, in fact, has become a component of clinical prediction rules that estimate risk for cough for patients given ACE-inhibitors [16]. Nevertheless, in this study, overall compliance and tolerance of both drugs was better among Asians, and better with telmisartan compared to ramipril. These findings should allay fears that Asians may have lower tolerance for the doses of ramipril and telmisartan that were used in these studies to lower cardiovascular risk. Thus, it would be appropriate to use the same dose of these medications in both Asians and non-Asians.

Although Asians were indeed smaller in body size than non-Asians in both studies, there was no evidence that any of the drugs were tolerated less. Regulatory agencies require trials to present a summary of safety and effectiveness data by ethnicity but few publications comply with this requirement for reporting [17]. Large International studies like ONTARGET and TRANSCEND offer the advantage of wide representation from various ethnic groups, which can aid us in evaluating local applicability of the results. Publication of results in ethnic subgroups from these trials can help allay concerns regarding applicability.
